# Comparison of the Proximate Composition, Vitamins (Ascorbic Acid, α-Tocopherol and Retinol), Anti-Nutrients (Phytate and Oxalate) and the GC-MS Analysis of the Essential Oil of the Root and Leaf of *Rumex crispus* L.

**DOI:** 10.3390/plants8030051

**Published:** 2019-02-28

**Authors:** Oladayo Amed Idris, Olubunmi Abosede Wintola, Anthony Jide Afolayan

**Affiliations:** Medicinal Plants and Economic Development (MPED) Research Centre, Department of Botany, University of Fort Hare, Alice 5700, South Africa; owintola@ufh.ac.za (O.A.W.); aafolayan@ufh.ac.za (A.J.A.)

**Keywords:** proximate composition, mineral content, vitamins, anti-nutrient, essential oil composition

## Abstract

Medicinal plants are a pertinent and effective remedy, employed in indigenous healthcare systems by traditional healers. This study focused on proximate parameters, minerals, vitamins, anti-nutrients and essential oil of the root and leaf of the medicinal plant; *R. crispus*, using the standard food analysis techniques. The result reveals that the moisture content of the leaf (7.57 ± 0.40%) and root (7.59 ± 0.08%) was not significantly different. The leaf has a higher ash, crude fat, fibre and mineral content than the root, except the carbohydrate (57.74 ± 3.06%) and Ca (1190.0 ± 0 mg/100g) values which are quite higher in the root. Traces of phytate was found in the leaf (1.15 ± 0.74%) and root (1.38 ± 0.27%) of *R. crispus*. The highest value of retinol, ascorbic acid and α-tocopherol was found in dried leaf (1.29 ± 0.014 mg retinol/100g), fresh leaf (159.73 ± 26.77 mg ascorbic acid/100g) and fresh root (54.90 ± 0.39 mg α-tocopherol/100g) respectively. The principal compound in the essential oil of the leaf are; 5-Eicosene, (E)-, docos-1-ene, trans-5-Octadecene, tetradecane while those found in the root are; 1-Heptacosanol, 4-Methyloctane, ethylcyclohexane, eucalyptol, m-Xylene, octadecane, phytol, and tetradecane. The research reveals that *R. crispus* may not only be used for medicinal purposes but could also be suitable for a complementary diet.

## 1. Introduction

The use of medicinal plants for therapy and other uses has always been part of human culture. The first records of the use of herbal medicine being acknowledged dated back 5000 years in China. On the other hand, India’s Ayurvedic traditional herbal medicine is believed to have been practiced for more than 5000 years [1]. For centuries, people have been so close to nature (the environment) and they rely on its fauna and flora as a source of medicine and food [2]. The uses of a particular species of plant for a specific ailment are similar across a geographical region and today, a large population of certain countries still depends on herbal remedies for their healthcare needs. In South Africa, the use of herbal medicine as an alternative therapy is on the increase and propagated by the traditional healers and elders in the communities. The extraction of crude plant extracts by the traditional healers either by decoction, tincture, infusion or other means are common practice all over the world. However, the effectiveness and mechanisms of action of the plant extracts are still under scrutinization scientifically. Several studies, in the fields of ethnopharmacology and medical ethnobotany, stressed the significance of phytotherapy to people in the different regions of the world, irrespective of the current civilization [3].

Morris (2011) [4], attests that the use of medicinal plants is the most widespread cultural heritage and the most ancient form of treatment. It was estimated at the end of the 20th century that more than two billion people globally (34% of the world’s population) will rely on medicinal plants as their principal treatment for illness [3]. At the moment, there is a lack of exact appraisals concerning uses of medicinal plants as resources for the treatment of ailment across the globe. Therefore, quantitative estimations of herbal plants used by folklore medicine could be useful tools to extrapolate the consumption of medicinal plant resources, but there are no records [5,6,7].

Several floras, used traditionally for medicinal purposes, have been the target of researchers in the pursuit for therapeutically effective drugs. The medicinal properties of plants are the function of the non-nutrient and the nutrient constituents. Among the medicinal plant ethnobotanically reported in South Africa, *Rumex crispus* L. is well known and it has been used for ages in traditional medicine, for its various biological activities and therapeutic efficacy [8,9]. The aerial and underground part of the plant is commonly used in different parts of the world for the treatment of various diseases. The young leaves of the plant are eaten as salads, soups, or cooked as a potherb. It is less bitter compared to the mature leaves that have more oxalic acid. The plant has been reported to be used for the treatment of anti-inflammatory, antimicrobial, dermatology infections, gastrointestinal tract diseases (e.g., ascariasis), upper respiratory tract diseases (such as a sore throat and nasal sinuses), laxative, treat piles, and dysentery [8,9,10]. The bioactive substances of *R. crispus* are a reflection of the medicinal importance due to its phytochemical composition such as; flavonoids (isorientin, vitexin, orientin and isovitexin), lipids, vitamins, carotenoids, organic acids and minerals. The root of *R. crispus* is rich in anthraquinones glycosides (chrysophanol and emodin) [11]. Generally, the genus of Rumex is characterized to be a rich source of anthraquinones [12], polysaccharides, carotenoids, triterpenoids, tannins, stilbenoids, flavonoids, naphthalenes, and naphthalenes [13]. In a non-shell, the juice from *R. crispus* is either consumed fresh or cooked and it contains some nutritional and anti-nutritional components.

*R. crispus* also possess essential oil. Essential oil is a concentrated hydrophobic aromatic liquid, containing volatile aroma compounds from plant’s secondary metabolite. They are used for aesthetic, medicinal purposes and food seasonings. The important roles of essential oils to plants are for protection against viral, insect, fungal, bacterial and herbivore attack [14]. Essential oils are also referred to as volatile oil; they may oxidize on exposure to the atmosphere, which may result in a change in colouration. Hence, there is need to store extracted essential oil in a cool, dry place, inside an airtight amber glass container. Essential oils are complex natural compounds with a strong aroma, usually produced by odoriferous plants. Quite a number of essential oils have been extracted from various plants such as; clove, eucalyptus, lemon, peppermint, cedarwood, frankincense, rosemary, lavender, *R. crispus* and many more. The volatile oil possesses by aromatic plants, bears some bioactive compounds with different pharmacological activities. The study of the constituents of essential oils is still understudied but the knowledge of volatile oils constituents is of enormous significance in the area of development of novel drugs [15,16].

The soil type, texture and exposure of a plant to environmental hazard could influence the nutritional compositions, and growth is proportional to the available nutrients in the soil [17]. Sorrenti et al. (2016) [18] confirmed and supported by Paetsch et al. (2017) [19] that environmental conditions can lead to modifications of the physicochemical properties of soil, and influences plants nutritional and phytochemical composition [20]. Therefore, the objectives of this study were to identify and compare the total proximate, vitamins and essential oil composition, of the root and leaf of *R. crispus*.

## 2. Materials and Methods

### 2.1. Plant Materials Preparation

The leaf and the root of wild *Rumex crispus* were collected (32°47′1.23′′ S, 26°51′9.85′′ E) in late summer of 2017, identified, and the sample was deposited in Giffen’s herbarium (Idr-Med-2017/03), Department of Botany, University of Fort Hare. The essential oil was extracted from the fresh plant sample with Solvent Free Microwave Extraction (SFME) and Hydrodistillation (HD). Subsequently, the remaining of the collected plant material was dried in a controlled environment at room temperature. The dried plant sample was pulverized with an electric blender (Polymix PX-MFC90D Switzerland) and stored in an airtight dark bottle in the refrigerator at 4 ± 2 °C until use.

### 2.2. Reagents Used

The chemicals used for this study were of standard grade, sourced from Sigma-Aldrich and Merck (Johannesburg, South Africa). Chemical Safety Precautions were taken during the assays and the following reagents were used; diethyl ether, sodium sulfite (Na_2_SO_3_), octanol, acetone, sulfuric acid (H_2_SO_4_), NaOH, boric acid, hydrochloric acid, KMnO4, FeCl_3_ solution, oxalic acid, 2,6-dichlorophenolindophenol, ∝-∝′-dipyridyl reagent, ethanol, ∝-tocopherol, chloroform, acetic anhydride, trichloroacetic acid, n-hexane, ascorbic acid, retinol and methanol.

### 2.3. Determination of Proximate Parameters

#### 2.3.1. Determination of Moisture Content

Moisture content was determined using the method as described by AOAC (2016) [21]. An empty crucible was dried to a constant weight in an oven at 105 °C, allowed to cool in a desiccator and weighed (*W*_1_). The pulverized plant sample (2.0 g) was weighed (*W*_2_) in the crucible and dried at 105 °C until it attained a constant weight. The crucible containing the plant sample was allowed to cool in a desiccator and the weight (*W*_3_) was measured. The moisture content was calculated in percentage thus as:

$$\%\;Moisture\;content=\frac{W_{2}-W_{3}}{W_{2}-W_{1}}\times \frac{100}{1}$$

#### 2.3.2. Determination of Ash Content

The AOAC (2016) [21], method was used for the ash content assay. A heat-resistant porcelain crucible was dried in an oven for 10 min at 105 °C, cooled in a desiccator and dry weight (*W*_1_) was measured. Thereafter 2 g of the pulverized plant sample was measured in the porcelain crucible and reweighed (*W*_2_). The crucible with the sample was incinerated in a furnace (Furnace 62700, Barnstead-Thermolyne, USA), first at 250 °C for 1 h and 550 °C for 7 h to ensure proper ashing. The crucible was removed, allowed to cool in a desiccator and weighed (*W*_3_). The percentage of ash content was thus evaluated as:

$$\%\;Ash\;content=\frac{W_{3}-W_{1}}{W_{2}-W_{1}}\times \frac{100}{1}$$

#### 2.3.3. Determination of Crude Fat

The crude lipid content of the sample was determinate by soxhlet extraction method as described by AOAC (2016) [21]. A pulverized sample of 5 g was measured in a 500 mL round bottom flask, containing a few grams of anti-bumping granules and was Weighed (*W*_1_) all together. The fat content of the pulverized plant samples was extracted in 100 mL of diethyl ether at 40–60 °C for 6 h in the flask attached to the Soxhlet extractor, at reflux. The filtrate was concentrated, diethyl ether was recovered and the oil in the round bottom flask was dried in an oven. The oil and the round bottom flask were thereafter Weighed (*W*_2_). The percentage of crude fat content was calculated thus as:

$$\%\;Crude\;fat=\frac{W_{2}-W_{1}}{Weight\;of\;Sample\;}\times \frac{100}{1}$$

#### 2.3.4. Determination of Neutral Detergent Fibre

The Neutral detergent fibre (NDF) of the samples was determined using the Van Soest et al. (1991) [22] fibre analysis. The pulverized, air dried sample (1 g) was weighed in an empty crucible (*W*_1_). At room temperature, 100 mL of neutral detergent solution and 0.5 g of sodium sulfite (Na_2_SO_3_) was added to the crucible, followed by few drops of octanol (octilic alcohol). The mixture was boiled for 60 min, filtered and the residue was washed twice in both boiling water and then in cold acetone. It was then dried in an oven at 105 °C for 8 h, cooled off in a desiccator and weighed (*W*_2_). The percentage of the neutral detergent fibre of the sample was calculated thus as:

$$\%\;NDF=\frac{(W_{1}+W_{2})-W_{1}}{Weight\;of\;Sample\;}\times \frac{100}{1}$$

where percentage Neutral detergent soluble (NDS): % NDS=(100−% NDF).

#### 2.3.5. Determination of Crude Protein and Nitrogen

The pulverized plant sample (2 g) was weighed in a 300 mL Kjeldahl flask and digested by a volume of 20 mL concentrated sulfuric acid (H_2_SO_4_). The process was aided with a catalyst until a clear solution was obtained. The digest was allowed to cool, diluted with 250 mL distilled water and transferred into a 500 mL Kjeldahl flask, containing anti-bumping chips and 40 mL of 40% NaOH. The distillate from the solution was transferred in a collecting liquid (250 mL of 2% boric acid and a few drops of mixed indicator) by immersing the end of the condenser inside the liquid to trap the gaseous ammonia being liberated. The resultant liquid was then back-titrated against 0.01 M hydrochloric acid until the endpoint violet colour was reached and percentage nitrogen content was calculated as:

$$\%\;W_{n2}=\frac{14\times M\times V_{t}\times V_{100}}{Weight\;of\;Sample\;\left(mg\right)\times V_{a}}$$

Percentage crude protein was expressed as: % *W_p_* = % *W_n_*_2_ × 6.25; Where *M* is actual molarity of acid (HCl), *V*_100_ is the titre value (Cm^3^) of HCl used, *V_t_* is the total volume of the diluted digest, *V_a_* is the aliquot volume distilled, *W_p_* is the crude protein content, *W_n_*_2_ is the Nitrogen content.

#### 2.3.6. Non-Fibre Carbohydrates

The Non-Fibre Carbohydrates (NFC) content of the plant sample was evaluated by the method described by Unuofin et al. (2017) [23]. The carbohydrate content was calculated by the difference of the total dry matter and the addition of the percentage ash, crude fat, crude protein and Neutral detergent fibre (NDF) using the formula:

%NFC=100−(%Ash+%Crude fat +%Crude protein +%NDF)

A high value of NFC indicates more digestible carbohydrates and lesser fibre in the sample.

#### 2.3.7. Determination of Energy Content

The value of energy in the samples was estimated in kilojoule per hundred gram and calculated by adding up the values for carbohydrate, crude lipid and crude protein using the factors; 16.736 KJ, 37.656 KJ and 16.736 KJ respectively as shown below:

$$\begin{array}{l} Energy\;value\;\left(KJ/100g\right)\\ \;=\left(\%Crude\;protein\;\times \;16.736\right)+\left(\%crude\;fat\;\times \;37.656\right)\\ \;+\;\left(\%carbohydrate\;\times 16.736\right) \end{array}$$

#### 2.3.8. Determination of Minerals

The method described by Bouba et al. (2012) [24], with modification was adopted. The pulverized plant samples (2.5 g) were weighed and ashed at 550 °C. The resulting residue (white-ash) was dissolved in 4 mL of concentrated HCl acid, filtered and the filtrate was diluted in a volumetric flask with distilled water. The resulting solution of the extract was then subjected for the analysis of specific major minerals. The analyses were performed in triplicates. The Iron (Fe) content was determined using Inductively Coupled Plasma-Optical Emission Spectrometer (ICP-OES; Varian 710–ES series) while the content of other elements; K, Na, Ca, Mg, Mn, Zn, Cu and P were determined by atomic absorption spectrophotometer (Varian Spectra AA-220, USA).

### 2.4. Quantification of Anti-Nutrient and Vitamins

#### 2.4.1. Determination of Oxalate Content

Oxalate content of the samples was determined by titration method as described by Unuofin et al. (2017) [23]. The samples (1 g) were weighed in triplicate in an Erlenmeyer flask which contains 75 mL of 3 M sulfuric acid. The mixture was stirred with a magnetic stirrer for 1 h, filtered and 5 mL of the filtrate was titrated against 0.05 M KMnO_4_ solution until a reddish brown colour persists. The oxalate content of the sample was expressed as 2.2 mg oxalate as an equivalent to 1 mL of 0.05 M of KMnO_4_.

#### 2.4.2. Determination of Phytic Acid

The phytic acid content of the sample was determined by the procedure described by Aina et al. (2012) [25]. To a 250 mL Erlenmeyer flask, 100 mL of 2% HCl was used to macerate 2.0 g pulverized plant sample for 3 h and then filtered. A volume of 25 mL of the filtrate was pipetted into 5 mL 0.3% ammonium thiocyanate solution in a conical flask. The solution was titrated against standard FeCl_3_ solution (0.001 95 g of Fe/mL) until an endpoint brownish-yellow colour persisted for a few minutes. The percentage of the phytic acid content in the sample was calculated thus as:

% Phytic acid=titre value×0.00195×1.19×100

#### 2.4.3. Ascorbic Acid (Vitamin C)

The ascorbic acid of the sample was quantified spectrophotometrically with modification as described by Njoku et al. (2015) [26]. The vitamin A content of both dry and fresh samples of *R. crispus* was tested, by weighing 1 g of plant sample in a flask containing 20 mL of 0.4% oxalic acid. The mixture was allowed to incubate for 5 min at room temperature and filtered. An aliquot of the filtrate (100 µL) was measured in a microtiter plate and 200 µL of 0.8 mg/mL of 2,6-Dichlorophenolindophenol was added. It was incubated for 5 min and the absorbance of the triplicates was taken at 520 nm using a stand-alone microplate reader (Diagnostic Automation, INC DAR800, USA). The standard solution (Ascorbic acid) was prepared similarly with concentration gradient (0.2, 0.4, 0.6, 0.8 and 1.0 mg/mL) and the results were expressed as equivalents of mg ascorbic acid per 100 g plant sample, using the equation from the standard curve: y = −0.698ln(x) − 0.0548, R^2^ = 0.935.

#### 2.4.4. Tocopherol (Vitamin E)

The Vitamin E content of the samples was evaluated as described [26]. The samples were weighed (1.0 g), macerated with 20 mL of absolute ethanol for 3 h and filtered. The filtrate was added to 0.2% ferric chloride (dissolved in 99.9% ethanol) in ratio 1:1 (*v*/*v*). An aliquot of 200 µL of the resulting solution was measured in a microtiter plate and 100 µL 0.5% of ∝-∝′-dipyridyl reagent in ethanol was added, the solution was then diluted with 50 µL distilled water. The absorbance of the triplicates was taken at 520 nm using a microplate reader. The standard ∝-tocopherol was prepared 100 mg/100 mL in absolute ethanol and graded (0.2, 0.4, 0.6, 0.8 and 1.0 mg/mL). Standard curve was plotted and vitamin E equivalent (mg d-alpha-tocopherol/100 g) was extrapolated from the standard curve gradient; y = 1.9283x − 6.2896, R^2^ = 0.9661.

#### 2.4.5. Retinol (Vitamin A)

The pulverized sample (1.0 g) was macerated in 20 mL of petroleum ether for 1 h. The mixture was filtered and evaporated to dryness. A volume of 0.2 mL solution of chloroform-acetic anhydride (1:1, *v*/*v*) was added to the residue, followed by 2 mL of (30%, *w/v*) Trichloroacetic acid (TCA) dissolved in chloroform, was added. The spectrophotometric absorbance was measured at 620 nm. Retinol standard was prepared in like procedure with gradient concentrations and the vitamin content of the sample was quantified to the retinol equivalent in micrograms (mg) retinol per 100 g, using the standard curve; y = 0.4744x − 0.1947, R^2^ = 0.9589.

### 2.5. Extraction of Volatile Oil (Essential Oils)

#### 2.5.1. Hydrodistillation Extraction (HDE) and Procedure

Freshly collected root and aerial part (400 g each) of *R. crispus* was subjected to hydrodistillation process for 3 h using Clevenger apparatus, following the procedure in accordance with the European Pharmacopoeia. The essential oil was extracted with 4 L of distilled water until there was no more essential oil in the sample. The essential oils were collected and stored in airtight amber bottles at 4 °C until it component was analysed.

#### 2.5.2. Solvent Free Microwave Extraction (SFME) of Essential Oils

SFME was performed using a laboratory microwave oven (Milestone Microwave, Apollo Scientific, South Africa). The microwave has a 2.45 GHz multimode reactor with a maximum power of 900 W which is delivered in an increment of 10 W with 650 nm wavelength. The power, time, temperature, and pressure of the microwave oven was digitally controlled by software (Easy-Wave) during the extraction and parameters were set at an optimum level to maximize the yield of essential oil. The SFME was carried out at standard atmospheric pressure, 400 g of the fresh plant samples were heated without any solvent. The extracted essential oil was separated by simple decantation. It was then dried under anhydrous sodium sulphate (Na_2_SO_4_), stored in airtight amber bottles at 4 °C until its component was subsequently analysed.

#### 2.5.3. Gas Chromatography-Mass Spectrometry (GC-MS)

The component analysis of the volatile oil was done on a gas chromatography (GC) and mass spectrometry (MS). The essential oil samples were prepared by diluting the extracted volatile oil with n-hexane (1:100, *v*/*v*). Analytical GC was carried out on a Chemetrix (Pty) Ltd.; Agilent Technologies 7890B GC System, ChemStation Rev. A.05.04 data handling system, equipped with a single injector and double flame ionisation detectors (FIDs). The operating conditions for the system during this analysis were set as follows; silica capillary columns with stationary Zebron (ZB) phase, ZB-5MS (5% Phenyl Methylpolysiloxane, 30 m × 0.25 mm × i.d., film thickness 0.25 μm). The temperature was set to 70 °C with a raising rate of 3 °C for 1 min, then held at 220 °C for 15 min and ramped for 3 min at 4 °C. The injection was initiated by 1 mL splitless injector with an initial temperature of 280 °C; carrier gas was GC grade helium adjusted to a linear velocity of 30 cm/s. The oven and detector temperature were set 280 °C; pressure at 8.27 psi; flush out time of 0.20 min and flows at the rate of 30 mL/min. MS (Agilent 5977 MSD) operating condition; source temperature was 230 °C; quadrupole temperature was 150 °C; the ionisation current, energy and mass was 60 µA, 70 eV and 35–350 µ respectively and the scan rate was 4.5 scans s^−1^.

#### 2.5.4. Identification of Components

The chemical components of the essential oils were identified by the GC-MS system by comparison of the mass spectra fragmentations, GC retention times, retention indices and percentage area composition of the compounds with the robust Library references (National Institute of Standards and Technology (NIST) Library 2014) by autonomously linear interpolating the component to the retention of the series of n-alkanes in database. The identification was further confirmed by searching through the NIST database. However, Chen et al. (2014) [27], suggested the combined application of GC-MS and nuclear magnetic resonance (NMR) spectroscopy may identify a more comprehensive chemical compounds than a single platform alone.

### 2.6. Statistical Analysis

Statistical analysis of the mean result was done using 1-way-ANOVA of variance and it was expressed as mean ± SD for samples collected. Significant differences between data were tested at 5% and 1% probability level, using Duncan multiple range test with Minitab 17, statistical software, and the significance of *p*-values was also determined.

## 3. Results

### 3.1. Proximate Composition

**Figure 1:**
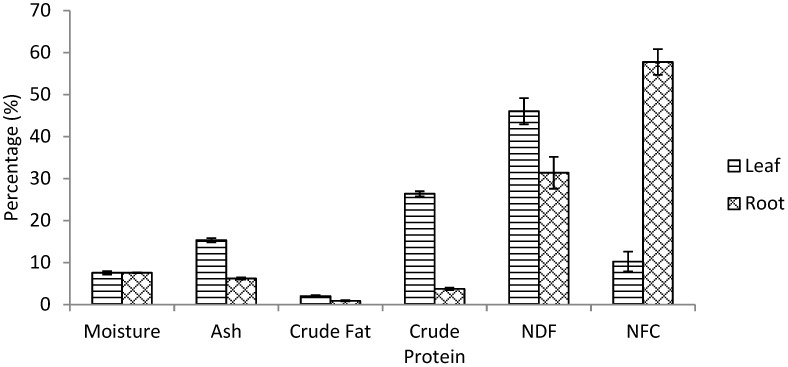
Percentage nutritional composition of the leaf and root of *R. crispus.* Non-Fibre Carbohydrates (NFC), Neutral detergent fibre (NDF).

The proximate composition of the dried pulverized root and leaf of *R. crispus* was evaluated and presented in Figure 1. The moisture content of the leaf and root (7.57 ± 0.40% and 7.59 ± 0.08% respectively) was not significantly different. The ash content of the leaf (15.35 ± 0.04%) was found to be higher than the root (6.22 ± 0.28%), which indicates a higher mineral concentration in the leaf. The level of fat in the leaf (2.01 ± 0.24%) was twice that of the root (0.90 ± 0.14%) and the crude protein of the leaf (26.37 ± 0.06%) was found to be significantly higher than the protein of the root (3.75 ± 0.31%). The fibre (NDF) level of the leaf (46.02 ± 3.13%) was higher than the root while the carbohydrate (NFC) of the root (57.74 ± 3.06%) was higher than the leaf, maybe due to lesser fibre levels (Figure 1). The estimated oxidizable energy of the root (1063.23 ± 95.31 KJ/100g) is significantly higher than that of the leaf (688.72 ± 81.5 KJ/100g), which could be due to the higher carbohydrate content of the root.

### 3.2. Mineral Composition

The mineral composition of *Rumex crispus* was measured and recorded in Table 1 below. The root has a higher composition of calcium (1190.0 ± 0 mg/100g), zinc (5.2 ± 0.14 mg/100g) and iron (32.95 ± 1.20 mg/100g) compared to the leaf; 635.0 ± 7.01 mg/100g, 4.10 ± 0 mg/100g, and 28.70 ± 0.70 mg/100g respectively. However, the magnesium (445.0 ± 7.00 mg/100g), potassium (5600.0 ± 0 mg/100g), sodium (280.0 ± 42.43 mg/100g), phosphorus (515.0 ± 7.01 mg/100g), copper (1.15 ± 0.7 mg/100g), and manganese (10.95 ± 0.21 mg/100g) composition of the leaf is significantly higher than that of the root as shown in Table 1.

### 3.3. Anti-Nutrient and Vitamins

The vitamin and the anti-nutrient compositions of the plant sample were quantified and presented in Table 2. The estimated retinol equivalent in the fresh plant sample was 1.22 ± 0.047 mg retinol/100g (Leaf) and 1.24 ± 0.018 mg retinol/100g (Root). The retinol per 100 g of the dry sample was estimated at 1.29 ± 0.014 mg (Leaf) and 0.74 ± 0.034 mg (Root). The ascorbic acid equivalent per 100 g was found to be higher in the fresh samples of the leaf and the root; 159.73 ± 26.77 mg and 43.07 ± 3.49 mg respectively while the ascorbic content of the dry sample of the leaf (26.73 ± 4.46 mg) and root (7.69 ± 1.35 mg) was lesser. The value of the Vitamin E content per 100 g of the fresh samples; 53.87 ± 0.21 mg (Leaf), 54.89 ± 0.39 mg (Root) and per 100 g of the dry samples; 54.71 ± 0.38 mg (Leaf), 54.41 ± 0.34 mg (Root) were slightly different from each other. The percentage phytate composition of the leaf (1.15 ± 0.74%) was found to be lesser than the root (1.38 ± 0.27%) while there was a slight difference in the oxalate content of the root (0.11 ± 0.0033 mg/g) and leaf (0.15 ± 0.019 mg/mg).

### 3.4. Essential Oil

The identified chemical components of the extracted essential oil from *R. crispus* was done with HDE and SFME as shown in Table 3. The molecular formula, Kovats index and percentage composition of the compound were contained within the table for the proper identification of compounds.

## 4. Discussion

Medicinal plants are of great economic importance, especially in the area of health improvement [2] and they play a significant role in drug development. Using a metabolite profiling, studies show that some plants have phytochemicals that are highly medicinal [44] and could be influenced by the nutritional and the essential oil composition of the plant. The traditional usage of *R. crispus* suggests, it is a plant of high medicinal value and it has been proven to have anthelmintic, anti-inflammatory and antibacterial potentials due to its bioactive component, especially polyphenols and alkaloids [9]. In this study, our results highlight the key proximate chemical composition of *R. crispus* and its relationship to traditional medicine and health benefits.

The shelf life of medicinal plants depends on moisture, temperature and other environmental factors. It was argued that plant material with a level of moisture higher than 8% favours the invasion of insects and moisture content of plant materials higher than 15% stands the risk of having been contaminated with bacteria and fungi [45]. The outcome of this research reveals that the moisture content of the dried samples of the leaf (7.57 ± 0.40%) and root (7.59 ± 0.08%) of *R. crispus* have low moisture and could be preserved with little risk of insect and microorganism invasion, hence increasing its shelf life. The result of the moisture content was close to the moisture content of *Rumex dentatus* Linn. and *Rumex nepalensis* Spreng. as reported by Hameed and Dastagir (2009) [46]. The level of the ash contents of the plant suggested the availability of inorganic constituents which is subsequently confirmed by the considerably high content of mineral compositions. Referring to the percentage of ash, our values in the leaf (15.35 ± 0.04%) slightly doubled the values of the root (6.22 ± 0.28%) of *R. crispus* which is in accordance with those described by Hameed and Dastagir (2009) [46]. The ash content closely suggested the reason why the mineral level of the leaf is higher than the root, except for the calcium composition in the root (1190.0 ± 0 mg/100g) which is significantly higher. The leaf is no doubt a better source of minerals than the root. In general, minerals have been confirmed to be essential in human nutrition for overall physical and mental wellbeing.

The fat and oil in the plant was extracted and quantified as shown in Figure 1. The leaf (2.01 ± 0.24%) has more fat and it was twice the value of fat recorded in the root (0.90 ± 0.14%). Fat is one of the major component causing differences in the gross energy of various food substance, it yields over 9 Kcal/g compared to carbohydrates and proteins that yield about 5 Kcal/g. Nagao and Yanagita, (2010) [47], reported medium-chain triglycerides (MCTs) and medium-chain fatty acids (MCFAs) have a therapeutic advantage in preserving insulin sensitivity as found in animal models and patients with type 2 diabetes. The level of oil in *R. crispus* could contribute to its medicinal potency. Digestible carbohydrates are another source of energy in the diet. The higher NDF level of the leaf (46.02 ± 3.13%) could have been due to lesser NFC (10.25 ± 2.37%) but these were contrary in the root which has higher digestible carbohydrate (57.74 ± 3.06%) and lesser fibre (31.38 ± 3.79%). The estimated oxidizable energy of the root was found to be 1063.23 ± 95.31 KJ/100g which is signifcantly higher than that of the leaf (688.72 ± 81.5 KJ/100g) and the reason is not far-fetched from the higher level of digestible carbohydrate contained in the root as shown in Figure 1. Elhayany et al. (2010) [48], concluded in their research that a low carbohydrate diet could reduce the risk of cardiovascular diseases and control diabetes among obese patients. *R. crispus* could be advantageous in this regard as the leaf is predominantly being consumed as a vegetable.

In this research, the organic nitrogen content was used to express the crude proteins (Nitrogen × 6.25), the highest values were found in the leaf (26.37 ± 0.06%) as compared to the protein content of the root (3.75 ± 0.31%) of *R. crispus*. According to the guidelines for dietary protein intake, 0.8 g per kilogram of body weight (g/kg BW/d) daily was recommended for human regardless of age or sex [49,50,51], however, this recommendation does not consider changes in hormone levels, immunity, metabolism and ill-health. The leaf of the *R. crispus* could, therefore, be recommended as a good source of protein in the complementary diet.

Phytate is the free-acid form of myo-inositol hexakisphosphate (InsP6) and it is the primary storage form of phosphorus in many plant tissues, predominantly seeds and bran. Although, there have been few reports of phytate being found in other tissue of the plant aside from the storage tissues or reproductive organs [52]. The values of the phytate in the leaf and root of *R. crispus* were found to be 1.15 ± 0.74% and 1.38 ± 0.27% respectively, which was considered to be trace according to Alkarawi and Zotz, (2014) [52]. On the other hand, phytate has been confirmed to have effects on absorption and digestion of some minerals and impacts negatively on some protein and lipid utilisation in the body. This is possible due to its affinity to chelate with cations such as magnesium, calcium, iron, zinc, potassium and copper to form insoluble salts [53]. Oxalate is an anti-nutrient that occurs in many plants as calcium oxalate deposit, in the tissue. The leaf and root of *R. crispus* were evaluated to have a deposit of calcium oxalate (0.15 ± 0.019 mg/g and 0.11 ± 0.0033 mg/g) respectively. The biological roles of oxalate in the plant include calcium regulation, tolerance to heavy metals, protection against herbivores and helps in plant growth, but in human, it could form a larger kidney stone that can block the kidney tubule [54]. Although oxalic acid has low toxicity with a minimal lethal dose of 5 g for an adult [55], a recommended dose not higher than 2.5 g in foods was suggested to avoid toxic effect [56].

The fat-soluble vitamin A content of the plant was evaluated using retinol equivalent. The dried sample of the leaf (1.29 ± 0.014 mg retinol/100g) has the highest retinol and the dried root (0.74 ± 0.034 mg retinol/100g) has the lowest value of vitamin A which is commensurable to the recommended Average Requirement (AR) of vitamin A, by European Union (EU) for average body weight adult men (68.1 kg) and women (58.5 kg) with an established ARs values of 0.57 mg retinol equivalent (RE)/day and 0.49 mg RE/day respectively [57]. Vitamin C is another essential micronutrient that was quantified in this research using ascorbic acid (AA) equivalent. It is a strong aqueous-phase antioxidant that scavenges free radicals and reduces oxidative stress [58]. The fresh sample of the leaf (159.73 ± 26.77 mg AA/100g) and root (43.07 ± 3.49 mg AA/100g) has higher ascorbic acid content compared to the dried samples. Vitamin C is less stable to heat, hence this might result in the lesser value of the ascorbic acid content of the pulverized samples as shown in Table 2. According to Frei and Trabe (2001), the recommended dietary allowance (RDA) of vitamin C for adults (≥19 years) is 75 mg/day for a female and 90 mg/day for a male. *R. crispus* could be a complementary diet for the deficiency of vitamin C. Unlike ascorbic acid, vitamin E is a fat-soluble vitamin with the most potent lipid-soluble antioxidant in the plasma. The daily RDA for vitamin E is 8 and 10 mg/day α-tocopherol equivalents for adult women and men respectively [59]. In contrast to the value of vitamin C, the vitamin E content of the dried and fresh samples do not show heat or moisture dependence. The fresh sample of the root (54.90 ± 0.39 mg α-tocopherol/100g) has the highest value, followed by the dried leaf (54.71 ± 0.38 mg α-tocopherol/100g). The least value of tocopherol was found in the fresh leaf (53.87 ± 0.21). This could be due to the thermal stability of vitamin E at high temperature as reported by Työppönen and Hakkarainen (1985) [60], that maximum destruction of vitamin E will occur at 120 °C for 24 h.

The essential oils obtained using SFME and HDE were quantitatively and qualitatively similar, but some compounds found in HDE are not found in SFME and vice versa in the essential oil of the fresh root and leaf of *R. crispus.* For comparison of chemical components of the extracted essential oil, same weight (400 g each) of fresh samples were used throughout the extraction process, the microwave extraction was done in 30 min without adding any solvent, and Hydro-distillation extraction was performed for 180 min using distilled water. The principal volatile compound found in the leaf are 5-Eicosene; possessing antimicrobial activities [32], docos-1-ene, trans-5-Octadecene, and tetradecane which was reported by Kavitha and Uduman (2017) [42] as an antimicrobial compound. While in the root; 1-Heptacosanol was one of the principal components which was reported to be nematicidal, anticancer, antioxidant and antimicrobial [29], 4-Methyloctane; could be used as tumor biomarker [31], ethylcyclohexane, tetradecane, eucalyptol; it has antinociceptive and anti-inflammatory properties [35], m-Xylene; It can enhance antibacterial and antifungal activities [38], octadecane; was reported by Girija et al., (2014) [40], to be active against bacterial, phytol; was also reported as an antimicrobial, anticancer, anti-inflammatory, and anti-diureti compound [41]. However, their proportions depend on the type of isolation technique used, HDE was found to have more yield (Table 3). In conclusion, the principal volatile compound such as tetradecane, 1-Heptacosanol, octadecane, tetradecane, were also part of the composition of the analysis of essential oil collected from *R. crispus* as reported by Miyazawa and Kameoka, (1983) [61].

## 5. Conclusions

The chemical composition determines the physiological properties and medicinal value of a plant. The medicinal potential of *R. crispus* could have been due to the chemical composition and nutritional value as studied in this research. The result of the proximate indicates that the plant is mainly constituted by carbohydrates and then followed by protein, ash, moisture and fat. The high level of ash revealed the presence of high mineral composition in the plant. In the results, vitamins and anti-nutrients show significant differences (*p* < 0.05); it was observed between the samples studied as recorded in Table 2. The essential oil component of *R. crispus* also revealed differences in the parts of the plant used. In conclusion, with the rapid increase in the demand for natural products, natural medicine and complimentary diet foods, *R. crispus* is a potential source that could be explored.

## Figures and Tables

**Table 1:** Mineral composition of the leaf and root of *Rumex crispus* in mg/100g.

Mineral	Leaf	Root
Ca	635.0 ± 7.01	1190.0 ± 0
Mg	445.0 ± 7.00	275.0 ± 7.07
K	5600.0 ± 0	630.0 ± 0
Na	280.0 ± 42.43	50.0 ± 0
P	515.0 ± 7.01	195.0 ± 7.07
Zn	4.10 ± 0	5.2 ± 0.14
Cu	1.15 ± 0.7	0.50 ± 0
Mn	10.95 ± 0.21	3.65 ± 0.7
Fe	28.70 ± 0.70	32.95 ± 1.20

The values are expressed as means ± standard deviations of triplicate analysis.

**Table 2:** Vitamin (A, C and E) and anti-nutrient (Phytate and Oxalate) composition of *R. crispus.*

	Leaf		Root	
	Fresh	Dry	Fresh	Dry
Vitamin A (mg retinol/100g)	1.22 ± 0.047 ^c^	1.29 ± 0.014 ^a^	1.24 ± 0.018 ^b^	0.74 ± 0.034 ^d^
Vitamin C (mg ascorbic acid/100g)	159.73 ± 26.77 ^a^	26.73 ± 4.46 ^c^	43.07 ± 3.49 ^b^	7.69 ± 1.35 ^d^
Vitamin E (mg α-tocopherol/100g)	53.87 ± 0.21 ^d^	54.71 ± 0.38 ^b^	54.90 ± 0.39 ^a^	54.41 ± 0.34 ^c^
Phytate (%)	1.15 ± 0.74 ^b^		1.38 ± 0.27 ^a^	
Oxalate (mg/g)	0.15 ± 0.019 ^a^		0.11 ± 0.0033 ^b^	

All data were expressed as mean ± standard deviations of the triplicate experiment (n = 3). Means are compared across the row and are significantly different if they do not share a letter. a > b > c > d.

**Table 3:** Chemical composition of the essential oil of *R. crispus.*

			Percentage Composition				
			Leaf		Root		Biological Activities
**Compound ^a^**	MF ^b^	KRI ^c^	SFME ^d^	HDE ^e^	SFME ^d^	HDE ^e^	
**1,3-Dimethyl-5-ethylbenzene**	C_10_H_14_	1058.2	1.10	2.67	-	-	-
**1-Docosanethiol**	C_22_H_46_S	2512.0	5.00	1.00	-	-	Anticancer [28]
**1-Heptacosanol**	C_27_H_56_O	2948.0	-	-	6.38	4.24	Nematicidal, anticancer, antioxidant and antimicrobial [29]
**2,5-Dimethylethylbenzene**	C_10_H_14_	1086.9	-	-	2.33	6.02	-
**2-Azacyclooctanone**	C_7_H_13_NO	1123.0	1.02	2.44	-	-	CNS stimulant [30]
**3-Methyloctane**	C_9_H_20_	871.4	3.23	1.97	2.48	6.84	-
**4-Methyloctane**	C_9_H_20_	862.85	5.20	2.23	4.86	10.05	Act as tumor biomarker [31]
**5-Eicosene, (E)-**	C_20_H_40_	2285.0	25.1	31.73	-	-	antimicrobial activity [32]
**Cyclohexadecane**	C_16_H_32_	1880.0	1.98	-	-	-	-
**n-Decane**	C_10_H_22_	1015.0	0.40	1.66	-	1.88	Antifungal and Antibacterial [33]
**Docos-1-ene**	C_22_H_44_	2194.0	7.31	9.03	-	-	-
**Dodecane**	C_12_H_26_	1214.0	1.79	1.3		-	Enhances antifungal activity [34]
**Eicosane**	C_20_H_42_	2009.0	1.18	1.92	1.15	3.95	antibacterial, antifungal, antitumor and cytotoxic effects [33]
**Ethylcyclohexane**	C_8_H_16_	829.1	-	-	11.89	6.90	-
**Eucalyptol**	C_10_H_18_O	1031.0	-	-	7.01	9.39	Antinociceptive, anti-inflammatory agent [35]
**Hemimellitene**	C_9_H_12_	1016.0	1.10	2.35	-	-	-
**Hexadecane**	C_16_H_34_	1600.0	3.47	2.48	2.11	5.18	Biosurfactants [36,37]
**Isononane**	C_9_H_20_	864.90	0.10	2.79	-	-	-
**m-Xylene**	C_8_H_10_	872.5	4.68	3.02	5.28	10.37	Enhance antibacterial and antifungal activity [38]
**n-Hexadecanoic acid**	C_16_H_32_O_2_	1972.0	3.10	1.87	-	-	Anti-inflammatory, antioxidant, nematicide, antiandrogenic, 5-Alpha reductase inhibitor, and potent mosquito larvicid [39]
**Octadecane**	C_18_H_38_	1810.0	2.73	2.24	25.61	9.74	Antibacterial [40]
**o-Xylene**	C_8_H_10_	893.2	-	-	3.56	1.50	Antifungal, Antioxidant and antimicrobial [33]
**Phytol**	C_20_H_40_O	2138.0	1.10	5.52	10.16	5.52	Antimicrobial, anticancer, anti-inflammatory, anti-diureti [41]
**p-Xylene**	C_8_H_10_	860.0	4.82	3.50	-	-	
**Tetradecane**	C_14_H_30_	1413.0	4.85	4.10	4.54	6.07	Antimicrobial activity [42]
**trans-5-Octadecene**	C_18_H_36_	1811.2	5.02	9.16	-	-	-
**Undecane**	C_11_H_24_	1115.0	2.85	1.24	1.12	4.22	-
**β-Cymene**	C_10_H_14_	1025.6	3.11	1.42	-	-	antioxidant, anti-inflammatory, antinociceptive, anxiolytic, anticancer and antimicrobial [43]
**Total percentage**			90.24	95.64	88.48	91.87	

^a^ Component separated in column; ^b^ Molecular formula; ^c^ Kovats retention index; ^d^ Solvent Free Microwave Extraction; ^e^ Hydrodistillation Extraction.
